# Dataset of thermal and visible aerial images for multi-modal and multi-spectral image registration and fusion

**DOI:** 10.1016/j.dib.2020.105326

**Published:** 2020-02-26

**Authors:** Lina M. García-Moreno, Jean P. Díaz-Paz, Humberto Loaiza-Correa, Andrés D. Restrepo-Girón

**Affiliations:** Universidad del Valle, Colombia

**Keywords:** Image registration, Image fusion, Image improvement, Thermal images, Visible images, Multi-modal, Multi-spectral, Photogrammetry

## Abstract

This article presents a dataset of thermal and visible aerial images of the same flat scene at Melendez campus of Universidad del Valle, Cali, Colombia. The images were acquired using an UAV equipped with either a thermal or a visible camera. The dataset is useful for testing techniques for the improvement, registration and fusion of multi-modal and multi-spectral images. The dataset consists of 30 visible images and their metadata, 80 thermal images and their metadata, and a visible georeferenced orthoimage. The metadata related to every image contains the WGS84 coordinates for allocating the images. Also, the homography matrices between every image and the orthoimage are included in the dataset. The images and homographies are compatible with the well-known assessment protocol for detection and description proposed by Mikolajczyk and Schmid [1].

**Table:** Specifications Table

Subject	Computer Vision and Pattern Recognition
Specific subject area	Visible and thermal images registration and fusion
Type of data	Images, text files, metadata and one orthoimage.
How data were acquired	*Thermal camera Zenmuse XT*,^1^*on board UAV Matrice 100*^2^ *Visible camera Zenmuse X3*,^3^*on board UAV Matrice 100*
Data format	Thermal images JPG format (336×256) and metadata KML format. Visible images JPG format (4000×3000) and metadata KML format. Georeferenced orthoimage.TIFF format (7437×7393), pixel size 3.5 cm, area 1.54 ha. Homography matrices ASCII files.
Parameters for data collection	Flying altitude and speed, and overlap ratio in function of the used sensor, whether *X3* or *XT*.
Description of data collection	Thermal images were captured using the *Zenmuse XT camera* on board of a *Matrice 100 UAV. Altitude = 100.4 m, Speed = 6.*4 m/s *and Overlap ratio = 90%.* Visible images were captured using the *Zenmuse X3 camera* on board of a *Matrice 100 UAV. Altitude = 80.9 m, Speed = 6.*4 m/s *and Overlap ratio = 80%.* *The* georeferenced orthoimage was generated using the captured visible images and the Agisoft Metashape^4^ software. The homography matrices were obtained from a small number of correspondences manually selected between the orthoimage and every thermal and visible image.
Data source location	Institution: Universidad del Valle. City/Town/Region: Cali/Valle del Cauca Country: Colombia. Latitude and longitude for collected samples/data: −76.5365°E 3.3785°N
Data accessibility	Repository name: Mendeley Data Data identification number: DOI: 10.17632/ffgxxzx298.1 Direct URL to data: https://data.mendeley.com/datasets/ffgxxzx298/1

**Table:** 

**Value of the Data** • This dataset presents thermal and visible aerial images of a flat scene with their respective geospatial data, a visible georeferenced orthoimage and the homography matrices between the images and the orthoimage, which are useful for other researchers to assess and develop new techniques for improvement, registration and fusion of thermal and visible images. • Using this dataset, researchers in fields of computer vision, remote sensing and pattern recognition can develop, improve and test matching methods between multi-modal and multi-spectral images. • The homography matrices included in this dataset can be used to assess new registration processes focused on images of different wavelengths, these homographies can also be used as an initial approximation to generate either visible or thermal orthoimages that allows fusion methodologies to be assessed.

## 1 Data

**Fig. 1:**
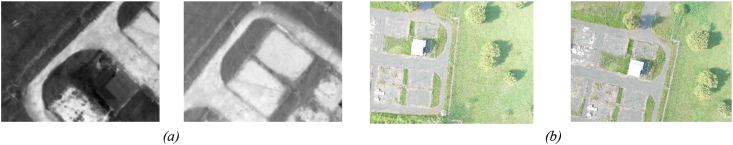
Four images of the dataset: (a) Two thermal images, (b) Two visible images.

**Fig. 2:**
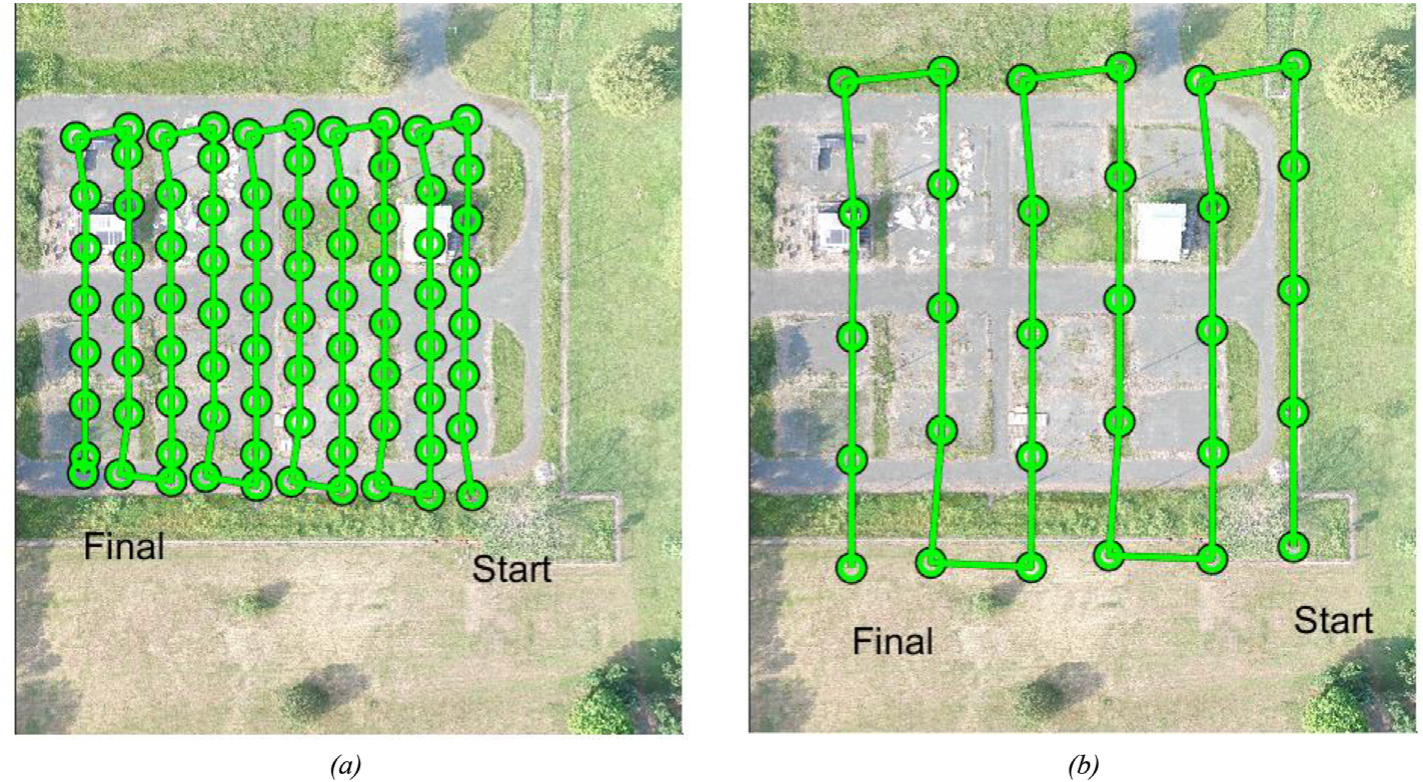
Photogrammetric flights for capturing (a) thermal and (b) visible images. The green lines illustrate the flight lines. The circle marks illustrate the images capture position. (For interpretation of the references to colour in this figure legend, the reader is referred to the Web version of this article.)

**Fig. 3:**
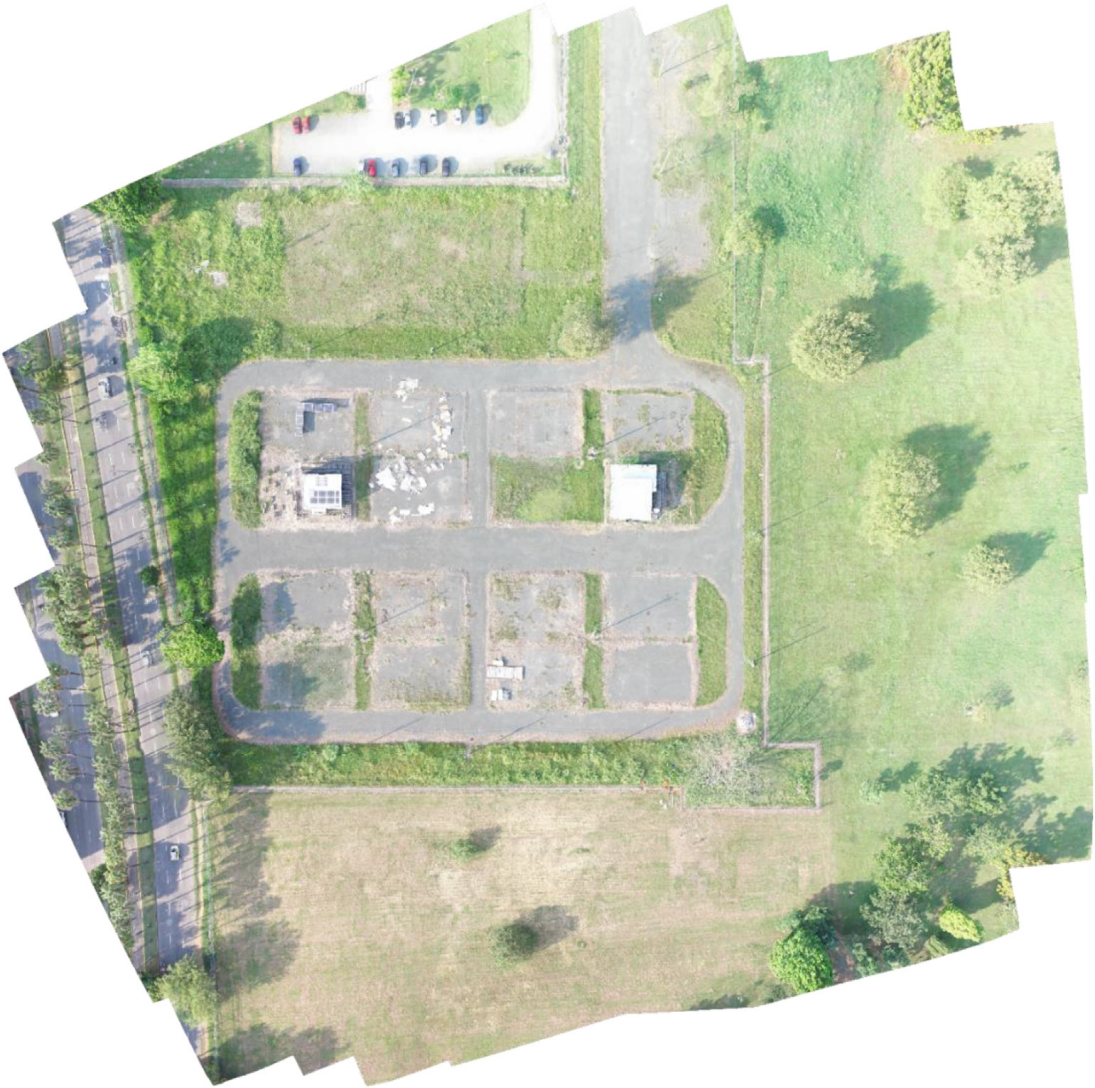
Visible orthoimage.

The dataset consists of 30 visible and 80 thermal images of a planar scene on an area of 1.54 ha at Universidad del Valle-Colombia (−76.536°E, 3.378°N). The images are compressed in JPG format and their WGS84 position is included to every header file. The dataset includes also one visible georeferenced orthoimage and the homography matrices between the orthoimage and every thermal and visible image. Table 1 presents the files and folders organization of the dataset. Table 2 and Table 3 presents the main specifications of the equipment used to capture the images of the dataset. Table 4 presents the approximate weather conditions while capturing the images. Fig. 1 shows two thermal and two visible images of the dataset. Fig. 2 shows the photogrammetric flights that were performed for capturing the images. Fig. 3 shows the visible orthoimage. Code 1 shows the Matlab^5^ function used to write the homographies between the images.

Code 1 Function for writing the homography matrix between two images

**Figure:**
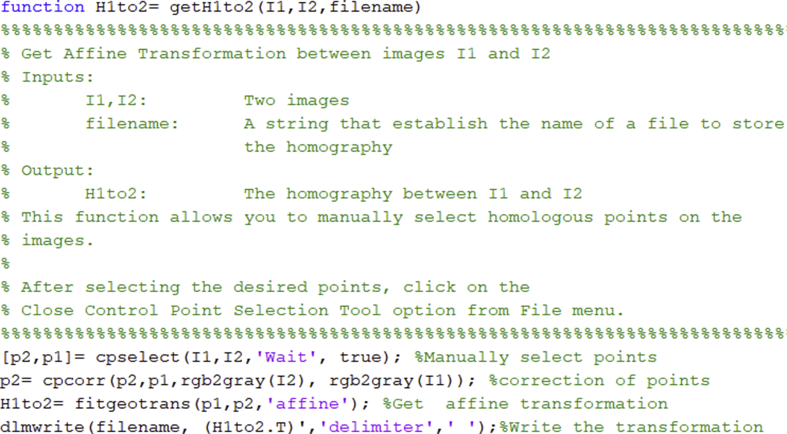

**Table 1:** Dataset organization.

Folder	Filename	Description
∖	ORTHOUV2018.TIFF	Visible georeferenced orthoimage.
∖THERMAL∖	LWIRXX.JPG	Thermal aerial images numbered as XX (01 − 80).
	HORTHOtoLWIRXX	Homography matrices between the orthoimage and the thermal aerial images.
	LWIR.kml	Geospatial data with WGS84 coordinates of capture position of the thermal images.
∖VISIBLE∖	VSXX.JPG	Visible aerial images numbered as XX (01 − 30).
	HORTHOtoVSXX	Homography matrices between the orthoimage and the visible aerial images.
	VS.kml	Geospatial data with WGS84 coordinates of capture position of the visible images.

**Table 2:** Cameras specifications.

Model	Spectral range (μm)	Image size (pixels)	Diagonal FOV (°)	Focal Length (mm)
Zenmuse XT	7.5–13	336 × 256	44	9
Zenmuse X3	0.4–0.7	4000 × 3000	94	20

**Table 3:** UAV Matrice 100 specifications.

Type	Hovering time full payload (min)	Max speed of ascent (m/s)	Max speed of descent (m/s)	Operating temperature (°C)
Quadcopter	20	5	4	−10 to 40

**Table 4:** Weather conditions while acquiring images.

Relative Humidity (%)	Weather Condition	Temperature (°C)	Wind speed (km/h)	Atmospheric Pressure (hPa)
55	Cloudly	29	19	1013

## 2 Experimental design, materials, and methods

For capturing the images of the database, the next materials and equipment were used:

• Zenmuse XT Thermal camera (Specifications at Table 2).
• Zenmuse X3 RGB camera (Specifications at Table 2).
• Matrice 100 UAV (Specifications at Table 3).
• DJI Go^6^ application software for planning flights.

First, the visible images were acquired in a photogrammetric flight with an approximate overlap ratio of 80% (∼28 m between consecutive images) for both longitudinal and transverse direction using the Zenmuse X3 camera on board of the Matrice 100 UAV flying at approximate altitude of 80 m and speed of 6.4 m/s, on Jan-17-2019 at 16:16 hours (GTM-5). Then, the thermal images were acquired in a photogrammetric flight with an approximate overlap ratio of 90% (∼12 m between consecutive images) for both longitudinal and transverse direction using the Zenmuse XT camera on board of the same UAV flying at approximate altitude of 100 m and speed of 6.4 m/s, on Jan-17-2019 at 16:40 hours (GTM-5). Fig. 1 shows two thermal images and two visible ones that were captured in their respective flights.

The photogrammetric flights for visible and thermal acquisition (See Fig. 2) were configured using DJI Go app. For the visible images, an approximate area of 1.57 ha was covered by 6 flight lines north-south orientated and separated by ∼28 m. The total flight length for capturing the visible images was 857 m. For the thermal images, an approximate area of 0.86 ha was covered by 10 flight lines north-south orientated and separated by ∼12 m. The total flight length for capturing the thermal images was 1009 m. The weather conditions when acquiring the images are listed in Table 4.

After capturing the visible images with their respective metadata, the Agisoft Metashape software generates a georeferenced orthoimage (See Fig. 3) that is a distortion-free representation with uniform scale over the complete scene. Agisoft uses SIFT for matching keypoints in a set of grayscale images and optimization algorithms to calculate the relative camera locations and a point cloud that allows reprojecting the images to generate the orthoimage that georeferenced in WGS84 [Semyonov, 2011].

The scene is considered to be mostly planar due to ∼9% of the visible orthoimage area was covered by 3D objects of more than 1 *m* height and the flights altitude for capturing the images was ∼80.9 *m*, for the visible ones, and 100.4 m, for the thermal ones. Therefore, the images can be related by homography matrices. The homography matrices between the orthoimage and every thermal and visible image are approximated as affine transformations. The homographies were computed using Code 1. It allows you to manually select at least 12 points between the orthoimage (reference image) and the thermal and visible images (target image). Then, it tunes the selected points using cross correlation. After, it approximates an affine transformation with the tuned points and saves the homography matrix. The obtained homographies are compatible with the well-known assessment protocol for detection and description that was proposed by Mikolajczyk and Schmid [1] and has been widely used to evaluate the performance of local descriptors with images on the same spectrum [[2], [3], [4]].

## Footnotes

1 https://www.dji.com/zenmuse-xt.

2 https://www.dji.com/matrice100.

3 https://www.dji.com/zenmuse-x3.

4 https://www.agisoft.com/.

5 https://www.mathworks.com/products/matlab.html.

## Footnotes

6 https://www.dji.com/goapp.
